# Political parties and climate policy: A new approach to measuring parties’ climate policy preferences

**DOI:** 10.1177/1354068817697630

**Published:** 2017-03-23

**Authors:** Neil Carter, Robert Ladrech, Conor Little, Vasiliki Tsagkroni

**Affiliations:** University of York, UK; Keele University, UK; University of Copenhagen, Denmark; Erasmus University Rotterdam, the Netherlands

**Keywords:** climate change, environmental politics, manifestos, party policy, political parties

## Abstract

This study presents an innovative approach to hand-coding parties’ policy preferences in the relatively new, cross-sectoral field of climate change mitigation policy. It applies this approach to party manifestos in six countries, comparing the preferences of parties in Denmark, France, Germany, Ireland, Italy and the United Kingdom over the past two decades. It probes the data for evidence of validity through content validation and convergent/discriminant validation and engages with the debate on position-taking in environmental policy by developing a positional measure that incorporates ‘pro’ and ‘anti’ climate policy preferences. The analysis provides evidence for the validity of the new measures, shows that they are distinct from comparable measures of environmental policy preferences and argues that they are more comprehensive than existing climate policy measures. The new measures strengthen the basis for answering questions that are central to climate politics and to party politics. The approach developed here has important implications for the study of new, complex or cross-cutting policy issues and issues that include both valence and positional aspects.

The success of the Paris Agreement, adopted at the COP21 climate conference in December 2015, depends heavily on the effectiveness of national climate change mitigation policies (henceforth climate policies). Political parties will play a critical role in determining whether governments develop these policies (Birchall, 2014; Jensen and Spoon, 2011; Schulze, 2014); they also have a unique role in shaping attitudes (Brulle et al., 2012); and they are central to our understanding of political risks and uncertainties in climate policy (IPCC, 2014: 6). This article addresses a lacuna in the literature by presenting an innovative approach to measuring the climate policy preferences of political parties that involves coding the climate change mitigation policy content of party manifestos.

Developing valid measures of parties’ climate policy preferences is a prerequisite for comparative research concerning issue politicization, party competition, party government and political leadership on climate change and we argue that existing measures, while useful, have important shortcomings, some of which are related to characteristics of climate policy itself. First, it is a relatively new policy area. While collecting data on new issues is obviously important, they can be difficult to incorporate into established coding schemes (Dolezal et al., 2014: 57). Second, climate policy is a cross-cutting and multisectoral issue, which makes it difficult to accommodate in hierarchically organized coding schemes. Third, climate policy may have both ‘valence’ and ‘positional’ aspects, which have implications for how it is measured (Carter and Clements, 2015; Gemenis et al., 2012). It shares these characteristics, to the varying degrees, with other issues such as social exclusion, European integration and immigration (Kriesi et al., 2008: 66; Guinaudeau and Persico, 2013; Castelli Gattinara, 2016: 18–20). We will argue further that existing attempts to measure parties’ climate policy preferences are limited by their relatively narrow focus on single countries, single parties and subsets of climate policies.

This study contributes to the nascent literature on parties’ climate policies by presenting a new approach that we apply to six countries, measuring the preferences of the two largest parties in Denmark, France, Germany, Ireland, Italy and the United Kingdom over the past two decades. Using Adcock and Collier’s (2001) types of measurement validation as a framework, we provide evidence for the measures’ validity through content validation and convergent/discriminant validation and we build on the existing research on parties’ environmental policy positions to develop a positional indicator of parties’ climate policy preferences.

The article begins by reviewing existing approaches to measuring parties’ environmental and climate policy preferences while setting out properties that valid measures of parties’ climate policy preferences should possess. It presents a new approach to comparing parties’ climate policy preferences and describes the coding of data from party manifestos. The analysis then examines the validity of the measures produced through content validation and convergent/discriminant validation, respectively, before assessing the validity of a positional measure of parties’ climate policy preferences. Finally, it discusses the strengths and weaknesses of the measures it produces, identifies questions to which they can be usefully applied and highlights the potential of this new approach for measuring party preferences in other policy areas.

## Measuring parties’ climate policy preferences

A climate policy is ‘a human intervention to reduce the sources or enhance the sinks of greenhouse gases’ (IPCC, 2014: 4). Climate policies therefore range across many substantive policy domains. There has been growing interest in national climate policies in recent years as a subject that is distinct from environmental policy. However, comparative scholarship on the *domestic politics of climate change* is relatively underdeveloped (Bernauer, 2013; Lachapelle and Paterson, 2013: 548) and political parties’ climate policy preferences, including their measurement, have received little attention.

Most measures of party preferences related to climate change focus on environmental policy, broadly construed. The Comparative Manifestos Project (CMP) (Budge et al., 2001; Klingemann et al., 2006) identifies and codes a diverse set of environmental issues in its ‘Environmental protection’ category (*per 501*). The Comparative Agendas Project (CAP) takes a similarly broad approach to coding environmental policy in party manifestos in its ‘Environment’ category. Significantly, it contains a subcategory (#705) that includes some important climate policy content (‘Air pollution, Global Warming and Noise Pollution’; henceforth CAP705) (Bevan, 2014). Several expert surveys include measures of parties’ environmental policy preferences (Bakker et al., 2015; Benoit and Laver, 2006; Rohrschneider and Miles, 2015). The expert-coded *EU Profiler* and *EU&I* data also includes parties’ positions on some specific environmental issues in 2009 and 2014 (Trechsel, 2009; Trechsel et al., 2014). Others have used relational content analysis of media coverage to measure parties’ preferences (Helbling and Tresch, 2011), including on the environment (Kriesi et al., 2008: 60).

Studies specifically addressing parties’ climate policy preferences are limited in their scope and comprehensiveness. Båtstrand (2014) examines the climate policies of four Norwegian parties in 2009, while Båtstrand (2015) provides a qualitative cross-national analysis of nine conservative parties. These studies identify climate policy pledges in party manifestos, but only if the party itself explicitly linked them to climate change. Moreover, Båtstrand’s interest is specific to certain research questions. The Norwegian study codes pledges only if they are relevant to the dimension underlying ‘old’ and ‘new’ politics (Båtstrand, 2014). The later, cross-national study, focuses on whether the parties ‘express trust in the concept of anthropogenic climate change’ and whether they propose climate policy measures ‘in line with free market environmentalism’ (Båtstrand, 2015).

Other studies focus on short periods in individual countries. De Blasio and Sorice (2013) compare the attention devoted to climate change by Italian parties in mid-2012, using keyword searches for ‘climate change’ and cognate terms in party documents. Case studies of individual parties (Carter and Clements, 2015) and studies of single-party governments also focus on parties’ climate policies (Birchall, 2014; Carter and Jacobs, 2014) but do not develop a systematic, general approach to measuring parties’ policy preferences.

We develop and examine new measures of parties’ climate policy preferences using two of Adcock and Collier’s (2001) types of measurement validation: content validation and convergent/discriminant validation. *Content validation* refers to the relationship between the indicator and the ‘systematized concept’ and it is a necessary condition for establishing overall validity. In this regard, a first desirable property of any indicator is that *it should include key elements and exclude inappropriate elements* (Adcock and Collier, 2001: 538–539).

The most fundamental problem regarding the validity of the measures described above relates to content validation. Some clearly leave out important elements of climate policy (e.g. Båtstrand, 2014, 2015; De Blasio and Sorice, 2013): The CMP codebook did not mention climate change until 2014. Hierarchical coding schemes such as the CAP and CMP present a more general problem: while mutually exclusive, hierarchically organized categories enable these data sets to cover a wide range of policy domains, they invariably exclude important content because a piece of text can belong only to one category (e.g. climate policy *or* energy *or* agriculture). Consequently, the salience of issues cutting across many categories is likely to be underestimated (Guinaudeau and Persico, 2013) and some measures leave out important elements of climate policy, such as renewable energy and energy efficiency measures, that are contained in other categories.

Some measures have the opposite problem: they include elements that clearly fall outside any definition of climate policy. This is the case for all general measures of environmental policy preferences, whether from manifestos, expert surveys or media content analyses. The CMP Environmental Protection category refers, among other issues, to ‘Animal rights’ and a ‘great variance of policies that have the unified goal of environmental protection’ (Volkens et al., 2016). The CAP Environment subcategories are likewise wide ranging, including, for instance, Drinking Water Safety and Water Supply (Bevan, 2014). This problem also applies to some climate policy-specific indicators. CAP705 includes such issues as ‘noise pollution development, rules of upper decibel levels in public space, noise nuisance in kindergartens’ (Green-Pedersen and Mortensen, 2014: 20).

*Convergent/discriminant* validation concerns an indicator’s relationships with other measures. We expect measures of the same concept to be empirically associated (i.e. to converge) (Adcock and Collier, 2001: 540); this is a second desirable property of any new measure. Following from this, the closer the association of a given measure with parties’ climate policy preferences (rather than environmental policy preferences), the stronger the relationship should be with the measures of climate policy preferences developed here. Yet it should not be so strong (i.e. approaching identity) to suggest that the measures developed here add little or nothing to existing measures.

Drawing on the literature on *position-taking* in environmental policy, we identify a third desirable property of a valid measure of climate policy preferences: *that it can take into account policy preferences that directly subvert climate policy goals.* Even where a party proposes climate change mitigation policies, the effects of those policies could be undermined if it also proposes policies that would increase emissions, such as increased support for new coal-fired power stations. Identifying such measures helps to control for internal inconsistency in party policy that may arise from ‘greenwashing’, the kind of ‘cheap talk’ that can be mistaken for an indicator of a party’s policy preferences.

While environmental policy is widely regarded as ‘a classic valence issue’, this assumption is increasingly being questioned. Climate policy in particular is an issue sometimes characterized by sharp disagreement, which can range from climate change deniers questioning the very fundamentals of climate science to conflict over specific climate measures, such as expanding onshore wind power or the use of green taxes. Such tensions can underpin partisan divisions over climate change (Carter and Clements, 2015; Guber, 2013). More generally, saliency theory has been questioned (Dolezal et al., 2014); the value of measuring both salience and position has been highlighted (e.g. Guinaudeau and Persico, 2014); and the CMP has been criticized for failing to separate its indicators of salience and position (Dolezal et al., 2014: 61–62; Lowe et al., 2011: 133; cf. Volkens, 2007: 117). We do not settle these questions here, but we do build on Compston and Bailey’s (2013) concept of ‘anti-climate policy’ and Weale et al.’s (2000: 247–250) approach to constructing an environmental policy index to develop a measure that can be regarded as positional at the level of climate policy preferences.

## Coding parties’ climate policy preferences

Existing manifesto-based projects using hand-coding provide a basis for important elements of our coding scheme. Like the CMP, the CAP and Båtstrand (2014, 2015), we use parties’ main pre-election documents as the principal source of data (see Online Appendix A). The benefits of using these documents are well known: they set out the party’s official policy preferences, they are publicly available and amenable to ex post analysis and they are unlikely to contain only cheap talk.

Like the CMP and the CAP projects, we use quasi-sentences – ‘the verbal expression of one political idea or issue’ (Klingemann et al., 2006: 165) – as the unit of observation (see Online Appendix B). We also share their assumption that the proportion of a party document devoted to a particular type of content is related to its ‘salience’ for that party, which in turn reflects its policy preferences.

Unlike these projects, we focus on a single policy area (climate policy), anchored in a single hypothetical policy outcome (greenhouse gas [GHG] emissions). We assume that the relative simplicity of our coding scheme reduces coding error compared to more complex schemes covering numerous policy areas, consistent with criticisms of coding scheme complexity made by both architects and critics of the CMP (Budge, 2006: 84; Mikhaylov et al., 2012: 80).^1^ Moreover, its relative simplicity facilitates the coding of a cross-sectoral issue, building on previous approaches to coding EU issues in the CAP project (Guinaudeau and Persico, 2013).

We aim to reduce potential ambiguity in the coding scheme (and, thus, the likelihood of coding error) by explicitly articulating our coding categories, which follow from the definition of climate policy set out above. Our first substantive concern is with ‘pro-climate’ content: content that *indicates support for policies that would, if implemented, reduce GHG emissions or enhance GHG sinks*. Many such policies in developed economies are well mapped in standard accounts (e.g. Compston and Bailey, 2016). They typically include supports for energy efficiency, the reduction of emissions from specific sectors (e.g. energy, transport and agriculture), and overarching measures such as carbon pricing and the creation of institutions to govern climate policy. However, party documents are not simply lists of policy proposals: much text simply expresses a party’s general attitude or sentiment on an issue. Where this indicates support for emissions-reducing policies, it is also coded as pro-climate content. Examples include content acknowledging climate change as a policy problem and expressing support for climate change mitigation or for environmental protection that implicitly includes climate protection.

Coding was carried out by researchers with expertise in climate policy and with knowledge of each country. Hand-coding of manifestos facilitated the application of context-sensitive expertise at the level of individual quasi-sentences (Volkens, 2007: 117). This expertise is important for two reasons: first, because the coding of these categories is, in principle, context specific: the same policy in two countries may have a different significance. For example, building nuclear power capacity in a country that depends wholly on coal for electricity generation will reduce GHG emissions; building it in a country that depends wholly on renewable sources of electricity may increase emissions. Second, sometimes further research was required to establish the policy’s prospective impact on GHG emissions at the time the manifesto was published, and coders with expertise were well placed to carry out that research. An example was high-speed rail in the United Kingdom, which was ultimately coded as having an ambiguous effect on the UK’s emissions.^2^ While, in practice, many policies were coded similarly across contexts, the accommodation of context-sensitive expertise speaks to criticisms of manifesto-based data for being insufficiently sensitive to context (Franzmann and Kaiser, 2006; Mölder, 2016) and has a precedent in evidence-based expert-coding (Trechsel, 2009).

We aimed to minimize error further through central coordination and standardized procedures, drawing on lessons from other, larger hand-coding projects (Budge et al, 2001: Ch. 4; Volkens et al., 2009). Coders received a set of instructions (Online Appendix B) and a piece of correctly coded text as an example. Where difficult coding decisions arose, these were coded as such and then discussed and resolved with (and among) the authors, who coordinated the coding process. Some 69% of manifestos were double-checked by different coders. This was particularly intensive earlier in the coding process, as difficult coding issues were resolved and coding decisions standardized (see Volkens et al., 2009: 244). However, this did not amount to independent coding of manifestos by multiple coders and like other projects based on hand-coding, we face potential problems of reliability (Volkens, 2007: 118). Where doubts remained about an item, claims made in the party document regarding the emissions impact of a policy measure were taken into account (i.e. parties were given the ‘benefit of the doubt’).

A set of subcategories was developed to provide insights into the substantive content of the pro-climate text and as a means of systematically varying the content of our measures (see Table 1). To assign text to these substantive subcategories, each quasi-sentence was inductively labelled with a topic and then aggregated into broader, logically coherent categories. The aggregation of these labels fed back into the development of a codebook delimiting the categories (Online Appendix C). Coders also completed a questionnaire concerning basic document characteristics for each manifesto that we use later in the analysis (Online Appendix D).

**Table 1.** Pro-climate subcategories.

	Mean % of pro-climate content
Core subcategories	
Pro-environment	35.1
Pro-climate policy (other)	14.4
Pro-lower carbon energy	12.8
Pro-lower carbon transport	11.4
Pro-energy efficiency	6.9
Pro-carbon sinks	3.1
Non-core subcategories	
Planning	7.6
Agriculture and food	5.6
Waste	3.1
Anti-growth	0.03

*Note*: See Online Appendix C for detailed descriptions of these subcategories. *N* = 62. Two manifestos contained no ‘pro-climate’ content.

Following the same procedures, we laid the basis for a positional measure of climate policy preferences by identifying anti-climate content. Drawing on Compston and Bailey’s (2013) work on governments’ anti-climate policies and a broader definition of climate policy covering all policy measures that influence emissions (EBRD and GRI, 2011: 60), we identified content that *indicates support for policies that would increase GHG emissions or diminish GHG sinks*. It includes quasi-sentences that deny that climate change is a problem, oppose climate change mitigation policies or make specific policy proposals (e.g. opening a new airport) that would increase GHG emissions (Compston and Bailey, 2013: 147–148; see Table 2).^3^

**Table 2.** Document attributes and climate policy preferences.

		*N*	% pro-climate content		% Core pro-climate content	
			Mean	*p* Value	Mean	*p* Value
Acknowledges climate change	No	24	5.1	0.04	3.7	0.00
	Yes	40	6.6		5.8	
Commits to national climate goals	No	33	5.7	*0.16*	4.4	0.04
	Yes	31	6.4		5.7	
Climate change in front matter*	No	43	5.5	0.02	4.3	*0.01*
	Yes	19	7.3		6.6	

*Note*: *p* Values are for one-tailed *t*-tests. *p* values for tests assuming unequal variance are in italics.

*Two documents did not include front matter.

## Case selection

The data cover 64 parties-at-elections in six countries (Denmark, France, Germany, Ireland, Italy and the United Kingdom) from the mid-1990s until 2015. The manifestos vary in length. The Danish documents are particularly short: 338 quasi-sentences on average, compared to a mean document length of 1161 quasi-sentences across all coded documents.^4^ Occasionally, the main parties were electoral coalitions (e.g. in Italy in 2001 and 2006). Sometimes, a party’s manifesto also represented smaller parties belonging to their electoral coalition (e.g. the Danish centre-left in 2011); here, we assume that the preferences of the main coalition party are accurately represented in the document (see Online Appendix A for details).

The six West European countries selected have much in common: they are all long-standing EU member states; they each have an established environmental policy arena; and, with the exception of France, they are heavily dependent on fossil fuels. Within that universe, they are diverse along dimensions that may influence the structure of climate politics (although given the paucity of existing research our expectations are necessarily tentative). They encompass both leaders and laggards on climate policy; small and large countries; a range of public concern about climate change; a variety of GHG emissions profiles, measured by per capita emissions, the share of emissions from agriculture compared to fossil fuel use and the range of policy effort required for the 2012 and 2020 commitment periods. Overall, we expect inter-country differences to be relatively small given these important similarities, an expectation supported by analysis of variance tests on each of the measures, which show no statistically significant differences between country means.

The period covered encompasses several electoral cycles in each country (32 in total) allowing us to examine variation in climate policy preferences within parties over time. It begins before the Kyoto Protocol was agreed (1997) and after climate change had become a distinct policy problem for governments in the early 1990s.

Within each country, we focus on the two largest parties by vote share before each election.^5^ Due to their centrality to coalition formation, national policy and public opinion, these are parties of particular substantive importance and therefore of importance for the study of party government and political leadership on climate change. The selection of parties also limits diversity in key respects. Each party could expect to enter government in the short or medium term (i.e. they were ‘parties of government’). Consequently, they could anticipate having to solve emergent policy problems; variation in their responses to climate change is therefore interesting and, in the face of a clear policy problem such as climate change, potentially puzzling.

In each country, we cover periods when each party has been in government and in opposition and, in each country, the two parties fall on either side of the main left-right cleavage structuring the party system (the exception being the Irish party system). Following from the existing studies of parties’ climate policies (e.g. Batstrand, 2014, 2015), we expect left-of-centre parties to develop more progressive climate policy preferences than right-of-centre parties.

## Pro-climate content: General description

**Figure 1.**
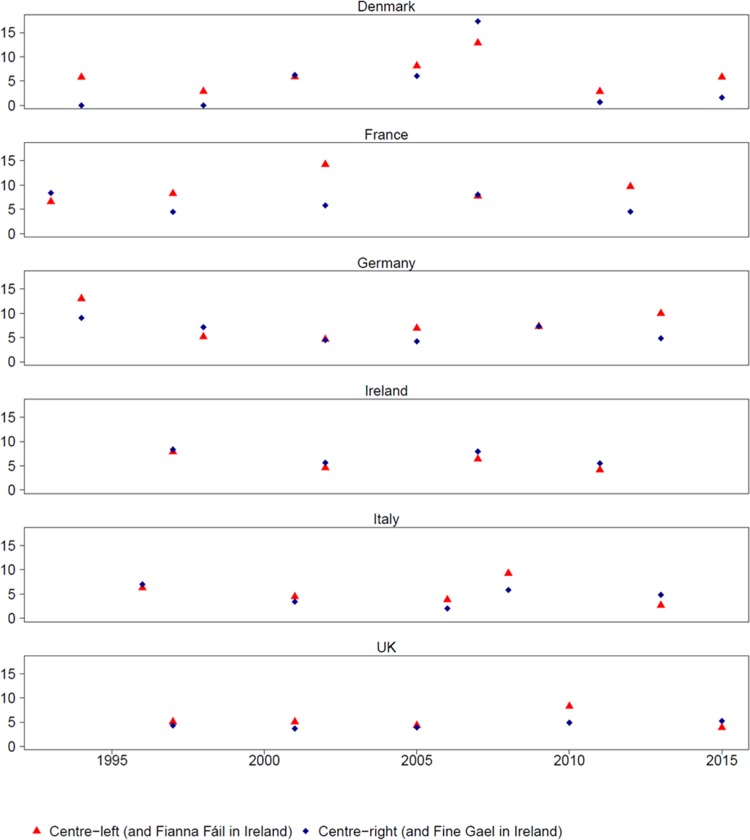
Pro-climate content.

Across 64 documents, 4568 quasi-sentences were coded as pro-climate content. The mean proportion of a manifesto accounted for by pro-climate policy is 6.0% (standard deviation (SD) = 3.1). Figure 1 shows considerable variation between parties and, within parties, variation over time. Denmark’s centre-right *Venstre*, for example, included no pro-climate content in 1994 or 1998, while in 2007, it occupied 17% of its manifesto’s text. This extreme case of within-party variation finds confirmation in case studies developed elsewhere (Seeberg, 2016). Other high points in the amount of pro-climate content (e.g. the Italian *Partito Democratico* in 2008; the Danish Social Democrats in 2007) also accord with existing case studies (Carter et al. 2014), as do some low points (the UK Conservatives in 1997 and 2001; Ireland’s Fianna Fáil in 2011; the Italian centre-right in 2006) (Carter and Clements, 2015; Little (2017); Pizzimenti, 2009). More generally, the difference between centre-left parties (mean = 6.8%) and centre-right parties (mean = 5.4%) is in the expected direction and statistically significant (*p* = 0.04), while the difference between pre-economic crisis (before mid-2008; mean = 6.4%) and parties since the crisis (after mid-2008; mean = 5.2%) is significant at the 0.1 level.^6^

## Content validation and a core measure

Perhaps the most fundamental difference between our data and alternative measures is the amount of content coded as relevant to climate policy and thus its comprehensiveness. The most directly comparable measure in the CAP (CAP705) includes an average of four quasi-sentences for each document we code. Both CMP Environmental Protection category (mean = 34 quasi-sentences) and the CAP Environment category (mean = 50) have a broader base of content. The content coded for our measure incorporates an average of 70 pro-climate quasi-sentences per document and is more squarely focused on climate policy *per se*.

Table 1 provides an overview of the substantive content of the text coded as pro-climate. In the average manifesto, 84% of pro-climate content is accounted for by six categories of quasi-sentence encompassing content that is generally acknowledged as being relevant to GHG emissions. These are general pro-environment content indicating support for reduced GHG emissions (35%) and content indicating support for lower carbon transport (11%), lower-carbon energy (13%), energy efficiency (7%), carbon sinks (3%) and other specific climate policy content (14%).

The remainder of the coded content, accounting for 16% of the average manifesto’s pro-climate content (and 1% of the manifesto’s overall content), concerns policies typically seen as being less central aspects of climate policy: planning, waste and agriculture measures, and negative mentions of economic growth. To address doubts concerning the relevance of the coded text in these categories, and following Adcock and Collier’s (2001: 539) advice to examine the effects of varying the content of indicators, we propose a second, core, measure that focuses on indications of support for a narrower set of core climate policies.^7^

## Convergent/discriminant validation

We assess the evidence for validity through convergent/discriminant validation in two parts. First, we examine the relationship between our measures of parties’ climate policy preferences and *document attributes* that serve as crude indicators of parties’ preferences. Second, we examine their relationship with *established measures* of parties’ environmental and climate policy preferences.

### Document attributes

We examine the following document attributes: whether the document acknowledges climate change as a problem; whether it commits the party to climate change targets; whether it mentions climate change in its front matter; and the number of mentions of climate change and cognate terms as a proportion of the overall word count. The relative frequency of these attributes appears to correspond to their significance as indicators of climate policy preferences: of the 64 documents, 40 acknowledge climate change as a problem, 31 make commitments to national climate change goals and 19 mention climate change in the document’s front matter.

We find strong evidence that these attributes are related to the general and core measures of parties’ climate policy preferences. For both measures and all of the document attributes, the difference in mean values is in the expected direction and, with one exception, these differences are statistically significant. The size of the mean differences (see Table 2) ranges from 0.7 to 2.3 percentage points, which, given that the general and core content accounts on average for 6% and 5% of manifesto content, respectively, seems sizeable. Climate change mentions (mean = 0.03) correlate positively and moderately with both measures. The correlation with the core measure (*r* = 0.45, *p* = 0.00) is stronger than the correlation with the general measure (*r* = 0.32, *p* = 0.01).

### Established measures

We also compare our measures to established measures of climate and environmental policy preferences for which data are available: the CAP climate policy and environment measures, the CMP Environmental Protection measure and its log-transformation devised by Lowe et al. (2011) and expert survey environmental salience measures. We expect positive correlations with each measure, but we do not expect the relationship to be so strong that they might be considered effectively identical. We also expect more specific measures of climate policy preferences (e.g. the CAP climate policy measure) to correlate more strongly than more general measures of environmental policy preferences.

The results in Table 3 bear out these expectations. The relationship between both general and core measures of ‘pro-climate content’ and four established salience-based measures of environment and climate policy is positive in all instances and is statistically significant (*p* < 0.05) in 8 of 10 instances. The correlations are moderate rather than strong and do not approach identity in any instance. They are stronger for CAP’s climate-specific measure than for the general environmental policy measures, with the exception of Lowe et al.’s (2011) measure. It is notable that the core measure correlates considerably more strongly with CAP705 than the general measure. The wide range of the expert survey correlation coefficients may reflect the small number of observations available for these data.

**Table 3.** Comparison with existing salience-based measures.

Data source	Issue	*N*	Measure	Pearson’s *r*	*p*
CAP	Climate*	34	General	0.42	0.01
			Core	0.54	0.00
CAP	Environment	34	General	0.29	0.1
			Core	0.39	0.02
CMP	Environmental protection	62**	General	0.4	0.00
			Core	0.48	0.00
Lowe et al. (2011)	Environment (importance)	50	General	0.46	0.00
			Core	0.54	0.00
Expert surveys***	Environment	24	General	0.42	0.04
			Core	0.32	0.12

*Note*: CAP: Comparative Agendas Project; CMP: Comparative Manifestos Project.

*CAP705. The available CAP data do not include Ireland or Germany.

**See Online Appendix E for details.

***Benoit and Laver (2006) and Bakker et al. (2015). See Online Appendix E for details.

## Positional measures

To develop a positional measure of climate policy preferences, we counterpose pro- and anti-climate content. For content validation, and in contrast to established positional measures, this has the merit of pitting two ‘opposites’ against one another, rather than two more loosely related concepts (i.e. environment vs. economy). Overall, 1971 quasi-sentences (2.7% of coded quasi-sentences or 31 per document, on average) were coded as anti-climate content. Despite our relatively conservative approach to coding anti-climate content (cf. Compston and Bailey, 2013), a large proportion of the substantive content of the anti-climate category consists of general economic policies (Table 4). These categories may contribute to a fuller picture of parties’ climate policy preferences, but they also risk ‘stretching’ the concept of climate policy (Sartori, 1970). At first sight, then, the relationship between this content and the concept of ‘climate policy preferences’ seems more tenuous than for the pro-climate category.

**Table 4.** Anti-climate subcategories.

	Mean % of anti-climate content
Core subcategories	
Pro-roads	8.6
Pro-aviation and shipping	6.2
Pro-fossil fuels	3.8
Anti-environmental taxes	3.4
Anti-climate (other)	1.8
Anti-nuclear	1.5
Non-core subcategories	
Pro-growth	32.5
Anti-taxes	18.6
Pro-tourism	10.4
Pro-global free trade	6.5
Agriculture	2.3

*Note*: See Online Appendix C for detailed descriptions of these subcategories. *N* = 62. Two manifestos contained no ‘anti-climate’ content.

To address this problem, we again identify two groups of quasi-sentences: core content referring to support for policies that are generally acknowledged as having a direct impact on GHG emissions and additional non-core content referring to more general economic policies.

To produce the general positional measure of parties’ climate policy preferences, we subtract the total anti-climate content from the total pro-climate content. This derives a mean climate policy position of 2.7 (SD = 6.2). Likewise, to produce a core positional measure, we subtract parties’ core anti-climate content from their core pro-climate content. The mean core position is 4.2 (SD = 3.5). The mean (absolute) difference between the general and core positional scores is 2.1 points (median = 1.3).

We again engage in convergent/discriminant validation by comparing these measures with document attributes and with established positional measures. The former comparison shows substantial and statistically significant mean differences in the expected direction (Table 5).

**Table 5.** Document attributes and climate policy preferences (positional).

		*N*	General climate policy position		Core climate policy position	
			Mean	*p* Value	Mean	*p* Value
Acknowledges climate change	No	24	0.1	*0.02*	2.6	
	Yes	40	4.2		5.2	0.00
Commits to national climate goals	No	33	1.2	*0.02*	3.3	
	Yes	31	4.2		5.1	*0.02*
Climate change in front matter*	No	43	1.4	*0.00*	3.3	
	Yes	19	5.6		6.1	0.00

*Note: p* Values are for one-tailed *t*-tests. *p* Values for tests assuming unequal variance are in italics.

*Two documents did not include front matter.

**Table 6.** Comparison with existing positional measures.

Data	Issue	*N*		Pearson’s *r*	*p*
EU Profiler/EU&I	Index: renewables and private transport taxation*	21	General	0.59	0.00
			Core	0.58	0.01
Environmental policy index (Weale et al., 2000)	Environment	62	General	0.48	0.00
			Core	0.44	0.00
Lowe et al. (2011)	Environment	50	General	0.34	0.02
			Core	0.29	0.04
Expert surveys**	Environment	32	General	0.39	0.03
			Core	0.46	0.01

*See Online Appendix E for details.

**Benoit and Laver (2006) and CHES (2010, 2014). See Online Appendix E for details.

General and core climate policy positions also correlate positively and significantly with four existing measures of parties’ environmental policy positions (Table 6): an additive index of two expert-coded positional climate policy items; Weale et al.’s (2000) environmental policy index using CMP data; a log-transformed measure proposed by Lowe et al. (2011) and positional items in expert surveys (Bakker et al., 2015; Benoit and Laver, 2006). These correlations are by far the strongest for the most climate-specific measure (almost reaching *r* = 0.6); for the general environmental policy measures, they range between 0.29 and 0.48.

## Discussion

Our analysis produces three sets of findings. First, regarding *content validation*, while the content of the pro-climate text tends to accord with existing knowledge concerning those policy categories most relevant to GHG emissions, the content of the anti-climate text as coded initially was less obviously related to the concept of climate policy. We responded by creating ‘core’ measures. Second, regarding *convergent/discriminant validation*, the measures are related to document attributes and to established measures of climate and environmental policy preferences. Their relationship with climate policy measures is markedly stronger than with environmental policy measures, suggesting that they are better measures of climate policy preferences than measures of general environmental policy preferences. Yet they do not come close to being identical with existing measures, suggesting that they constitute a new and distinctive contribution to the measurement of parties’ climate policy preferences. Contextual differences between parties (left-right differences, the presence of the economic crisis) and accounts of individual cases also converge with expectations. Third, we have developed *positional measures*, which also accord with our expectations concerning convergent/discriminant validation.

Not only are our measures empirically distinct from extant measures of parties’ environmental and climate policy preferences, the approach that produces them also has several advantages. It accommodates the cross-sectoral nature of climate policy; so, in common with Guinaudeau and Persico’s (2013) approach to EU policy, it can provide a model for studies of other cross-sectoral policy areas. The coding scheme is relatively simple and, based on existing arguments concerning coding scheme design, we assume that this minimizes error. The coding process allows for contextual specificity within a systematic framework for scoring cases, which enables its application to other contexts, including future party documents, while being based on a fixed assumption: that reducing GHG emissions will remain the central outcome in climate policy. It covers as many aspects of ‘climate policy’ as possible, as evidenced by the amount of content coded compared to other projects. In the ‘trade-off between parsimony and completeness’ (Adcock and Collier, 2001: 539), we argue that existing measures err on the side of parsimony, not least in the case of climate policy. Where there is doubt about the evidence from content validation, our coding of subcategories allows researchers to vary the content of the measures systematically without having to recode the texts themselves. Finally, in contrast to measures of salience, we produce a measure which aims to account for the positional aspect of climate politics and which may help to control for contradictions in party policy, including greenwashing.

These observations require at least two riders. First, our measurements should be regarded as ‘falsifiable claims’ (Adcock and Collier, 2001: 532). Second, we do not claim that existing approaches or data are without merit. The moderate-to-strong correlations with our measure indicate convergence, even if these measures evidently include content that is not relevant to climate policy or exclude content that is relevant to climate policy. Moreover, beyond their measurement of climate policy preferences, these approaches have further added value, such as including multiple other issues (CAP, CMP) and focusing on interesting theoretical questions (Båtstrand, 2014, 2015).

A question that we have not addressed directly is which of our four measures is ‘best’. Content validation – a prerequisite for overall validity – suggests there is doubt about our general positional variable, as elements of anti-climate policy may stretch the concept of climate policy. More generally, we show that ‘anti-climate policy’, while intuitive and useful, can be problematic in its application, even when applied conservatively.

Distinguishing between the merits of the other three measures (general, core and core positional measures) is more difficult. We have no ‘true’ measure of parties’ climate policy preferences against which they can be evaluated for criterion validity. The three measures take into account overlapping but somewhat different content (Tables 2 and 4). The relative merit of the positional measure may vary depending on how climate policy is conceived as an issue (valence or positional). We have highlighted arguments indicating the latter, but we do not regard them as definitive. The nature of the issue may vary between context and over time and it may be useful to measure both salience and position (Guinaudeau and Persico, 2014). Moreover, core and ‘non-core’ content as presented here is an informed approximation rather than a definitive distinction.

Significantly, our analyses show that binary indicators of document attributes discriminate between parties with stronger and weaker climate policy preferences – a potentially valuable insight highlighting measures of party policy preferences that can be collected at low cost.

We acknowledge that our approach has possible shortcomings. Although our positional measure has the merit of pitting two clearly articulated opposing concepts against one another, rather than the traditional ‘economy vs environment’ approach, it is not a ‘pure’ positional measure. This problem is difficult to avoid in manifesto-based approaches focusing on a broad policy dimension. In common with previous efforts to derive measures of policy preferences from manifestos, we weight each unit of content equally, whereas clearly some policies are more significant for GHG emissions than others. The main alternative is to estimate the ‘weight’ of various pieces of content in terms of GHG emissions; outside this approach, a climate policy expert survey may implicitly take this into account. Finally, although we explicitly focus on minimizing error (and maximizing validity) through the design of the coding scheme and mechanisms of control, standardization and cross-checking, we also acknowledge that using multiple independent coders is desirable and would allow us to measure that error.

## Conclusion

This article has presented an innovative approach to measuring parties’ policy preferences consisting of a set of salience and positional measures of climate change mitigation policy and has applied it to party manifestos in six European countries. It has presented evidence for the validity of these measures and has found that they are empirically distinct from and more comprehensive than extant measures. It argues that these measures represent a significant improvement on existing measures of parties’ climate policy preferences.

When new, cross-sectoral issues come on to the policy agenda and become increasingly distinct from established policy dimensions, parties’ preferences regarding those issues need to be measured so that questions central to party politics can be answered. The approach developed here can be extended to other policy areas and may be particularly beneficial for policies that are new, complex or cross-cutting or that include valence and positional elements. One example is immigration policy (Castelli Gattinara, 2016: 17–20; Kriesi et al., 2008: 66). While immigration is more regularly seen as a positional issue than climate change, it could benefit from anchoring its coding in two opposite policy outcomes (more vs. less immigration) and from the overall simplicity of a one-dimensional coding scheme. Other such issues may include European integration and social exclusion.

Measuring parties’ climate policy preferences is an important step towards understanding their development and how they might shape other outcomes, especially government policy. We hope that these measures will be taken forward and applied to questions that are central to climate politics and to party politics. This may lead to further evidence for the validity of these measures, corresponding to ‘nomological/construct validation’ (Adcock and Collier, 2001: 543) as hypothesised relationships (e.g. between party preferences and government policies or between economic conditions and party preferences) are confirmed. This kind of research can also contribute to the broader climate change research agenda and specifically to our understanding of the political obstacles to and opportunities for effective policy.

## Supplemental material

Supplemental Material, CarterEtAl_Climate_PP_appendixes_only - Political parties and climate policy: A new approach to measuring parties’ climate policy preferences Click here for additional data file. Supplemental Material, CarterEtAl_Climate_PP_appendixes_only for Political parties and climate policy: A new approach to measuring parties’ climate policy preferences by Neil Carter, Robert Ladrech, Conor Little, and Vasiliki Tsagkroni in Party Politics

## Supplemental material

Supplemental Material, CarterLadrechLittleTsagkroni_PP_Archive - Political parties and climate policy: A new approach to measuring parties’ climate policy preferences Click here for additional data file. Supplemental Material, CarterLadrechLittleTsagkroni_PP_Archive for Political parties and climate policy: A new approach to measuring parties’ climate policy preferences by Neil Carter, Robert Ladrech, Conor Little, and Vasiliki Tsagkroni in Party Politics

## Supplemental material

Supplemental Material, CarterLadrechLittleTsagkroni_PP_Codebook - Political parties and climate policy: A new approach to measuring parties’ climate policy preferences Click here for additional data file. Supplemental Material, CarterLadrechLittleTsagkroni_PP_Codebook for Political parties and climate policy: A new approach to measuring parties’ climate policy preferences by Neil Carter, Robert Ladrech, Conor Little, and Vasiliki Tsagkroni in Party Politics

## Supplemental material

Supplemental Material, pp-2016-online_appendix3 - Political parties and climate policy: A new approach to measuring parties’ climate policy preferences Click here for additional data file. Supplemental Material, pp-2016-online_appendix3 for Political parties and climate policy: A new approach to measuring parties’ climate policy preferences by Neil Carter, Robert Ladrech, Conor Little, and Vasiliki Tsagkroni in Party Politics

## Notes

1. We outline several ways in which we aimed to minimize error. We cannot measure the reduction in error resulting from these decisions; rather, our argument that these features reduce error is based on assumptions that are grounded in the existing literature.

2. ‘Ambiguous’ quasi-sentences were not counted as pro- (or anti-) climate content.

3. We use Compston and Bailey’s (2013: 148) list of anti-climate policies as a starting point, but we do not adhere to it strictly (see Online Appendix B).

4. We ran the tests for convergent/discriminant validation that follow while excluding the Danish documents (*n* = 14). Our findings are generally borne out by these tests, although in some instances the reduced *n* leads to higher *p* values (see Online Appendix F).

5. There is one marginal exception to this rule: Denmark’s Venstre before the 1994 election. In 1990, it had secured 0.6% less than the Conservatives.

6. One-tailed *t*-tests assuming equal variance.

7. The core measure developed here is unrelated to Jahn’s (2011) core measure of left-right preferences.
