# Supplementary material for: Political parties and climate policy: A new approach to measuring parties’ climate policy preferences
Source: Party Politics. 2017 Mar 23;24(6):731–42. doi: 10.1177/1354068817697630 (PMC6201165; doi:10.1177/1354068817697630)
Supplement: Supplemental Material, CarterEtAl_Climate_PP_appendixes_only - Political parties and climate policy: A new approach to measuring parties’ climate policy preferences [file CarterEtAl_Climate_PP_appendixes_only.docx]

**Online Appendices**

**Online Appendix A. Parties and documents.**

**Table A1. Party documents**

| **Country** | **Election** | **Centre-left party** |  | **Centre-right party** |  |
| --- | --- | --- | --- | --- | --- |
| **Denmark** | 1994 | Socialdemokraterne | *Det går bedre for Danmark.* | Venstre | *Det vil Venstre.* |
|  | 1998 | Socialdemokraterne | *Det kan gå to veje. Vores vei – Deres vej.* | Venstre | *Det vil Venstre.* |
|  | 2001 | Socialdemokraterne | *Mennesker Først: Fri og fælles i det 21. Århundrede.* | Venstre | *Tid for forandring.* |
|  | 2005 | Socialdemokraterne | *Mærkesager: Her kan du læse om de områder, hvor Socialdemokraterne vil gøre en ekstra indsats.* | Venstre | *Valgløfter.* |
|  | 2007 | Socialdemokraterne | *Vi vælger velfærd: Socialdemokraternes grundlag for folketingsvalget d. 13. November.* | Venstre | *Valggrundlag, Folketingsvalg 13. november 2007: Et endnu bedre samfund.* |
|  | 2011 | Socialdemokraterne | *Danmark skal videre.*** | Venstre | *Valggrundlag.* |
|  | 2015 | Socialdemokraterne | *Det Danmark du kender.* | Venstre | *Flere danske job i hele Danmark.* |
| **France** | 1993 | Parti socialiste | *Le contract pour la France.* | Union pour la démocratie française/ Rassemblement pour la République* | *Le 40 priorites de l’ UDF pour l’alternance /La France en mouvement. Rassembler pour changer.* |
|  | 1997 | Parti socialiste | *Changeons d’ Avenir.* | Union pour la démocratie française / Rassemblement pour la République* | *Programme du RPR et de l’UDF pour les elections legislatives de 1997.* |
|  | 2002 | Parti socialiste | *Programme pour les legislatives 2002.* | Union pour un mouvement populaire | *25 engagements pour la France, avec Jacques Chirac.* |
|  | 2007 | Parti socialiste | *Réussir ensemble le changement.* | Union pour un mouvement populaire | *Contrat de legislature 2007-2012.* |
|  | 2012 | Parti socialiste | *Le changement c’est maintenant.* | Union pour un mouvement populaire | *Projet 2012: Proteger et preparer l’avenir des enfants en France.* |
| **Germany** | 1994 | Sozialdemokratische Partei Deutschlands | *Reformen fuer Deutschland.* | Christlich-Demokratische Union/Christlich-Soziale Union* | *Regierungsprogramm von CDU und CSU.* |
|  | 1998 | Sozialdemokratische Partei Deutschlands | *"Arbeit, Innovation und Gerechtigkeit"*  *SPD-Programm für die Bundestagswahl 1998.* | Christlich-Demokratische Union/Christlich-Soziale Union* | *Wahlplattform von CDU und CSU.* |
|  | 2002 | Sozialdemokratische Partei Deutschlands | *Erneuerung und Zusammenhalt - Wir in Deutschland Regierungsprogramm 2002 – 2006.* | Christlich-Demokratische Union/Christlich-Soziale Union* | *Leistung und Sicherheit Zeit für Taten Regierungsprogramm 2002/2006 von CDU und CSU.* |
|  | 2005 | Sozialdemokratische Partei Deutschlands | *Vertrauen in Deutschland.* | Christlich-Demokratische Union/Christlich-Soziale Union* | *Deutschlands: Chancen nutzen.* |
|  | 2009 | Sozialdemokratische Partei Deutschlands | *Sozial und Demokratisch.* | Christlich-Demokratische Union/Christlich-Soziale Union* | *Wie haben die Kraft – Gemeinsam für unser Land* |
|  | 2013 | Sozialdemokratische Partei Deutschlands | *Das wir Entscheidet.* | Christlich-Demokratische Union/Christlich-Soziale Union* | *Gemeinsam erfolgreich für Deutschland.* |
| **Ireland**  ******* | 1997 | Fianna Fáil | *1997 General Election Manifesto.* | Fine Gael | *Securing and sharing our prosperity.* |
|  | 2002 | Fianna Fáil | *Election manifesto 2002.* | Fine Gael | *A Forward looking Ireland.* |
|  | 2007 | Fianna Fáil | *Now, the next steps.* | Fine Gael | *General election manifesto 2007.* |
|  | 2011 | Fianna Fáil | *Real Plan, Better Future.* | Fine Gael | *Let’s get Ireland working.* |
| **Italy** | 1996 | L’Ulivo | *Tesi per la definizione della piattaforma programmatica dell’Ulivo.* | Forza Italia | *Contratto con gli italiani.* |
|  | 2001 | L’Ulivo* | *Rinoviamo l’Italia, insieme. Il programma dell’Ulivo per il governo 2001/2006.* | Casa delle Libertà* | *Piano di governo per un’intera legislature.* |
|  | 2006 | L’Unione* | *Per il Bene Dell’Italia. Programma di Governo 2006-2011.* | Casa delle Libertà* | *Programma Elettorale.* |
|  | 2008 | Partito Democratico | *Un Italia moderna. Si puo’ fare.* | Popolo delle Libertà | *7missioni per il future dell’Italia.*** |
|  | 2013 | Partito Democratico | *L’Italia Giusta. Programma.* | Popolo delle Libertà | *Noi Ci Impegniamo. Programma. Elezioni Politiche 24 25 Febbraio.*** |
| **UK** | 1997 | Labour | *New Labour because Britain deserves better.* | Conservatives | *You can only be sure with the Conservatives.* |
|  | 2001 | Labour | *Ambitions for Britain. Labour’s manifesto 2001.* | Conservatives | *2001 Conservative Party General Election Manifesto. Time for Common Sense.* |
|  | 2005 | Labour | *Britain forward not back. The Labour Party manifesto 2005.* | Conservatives | *Are you thinking what we’re thinking? It’s time for action.* |
|  | 2010 | Labour | *The Labour Party Manifesto 2010. A future fair for all.* | Conservatives | *Invitation to Join the Government Britain. The Conservative Manifesto 2010.* |
|  | 2015 | Labour | *2015. Britain can be better.* | Conservatives | *Strong leadership, a clear economic plan and a brighter, more secure future.* |
| * = electoral coalition, ** = programme of a broader electoral coalition, *** = both parties are centre-right | | | | | |

**Online Appendix B. Coding instructions.**

*This is an abridged and annotated version of instructions sent to coders in December 2014. It excludes some variables relating to policy domains that we did not carry forward into the coding of all manifestos and which are not relevant to this study. These instructions were supplemented by a correctly coded piece of text, by more detailed and context-specific discussions with the authors about individual pieces of content/policies, and by a guide to identifying quasi-sentences from Froio (2012). Existing research indicates that using sentences would have no significant impact on our measures’ validity (Däubler et al., 2012), but we follow the CMP and most national CAP projects in using quasi-sentences for practical reasons: for four countries we had access to the quasi-sentences used by the CAP.*

There are four basic coding categories for quasi-sentences: 1. Pro-climate policy; 2. Anti-climate policy; 3. Neutral; 4. Not sufficiently relevant. They correspond to codes for the ‘ccode’ (climate code) variable.

**1. Pro-climate policy quasi-sentences**

These are pieces of text that *indicate support for policies that, if implemented, would reduce net greenhouse gas emissions or increase carbon sinks*.^^[[1]](#footnote-2)^^ These quasi-sentences may be general statements that acknowledge climate change as a policy problem (e.g., ‘Climate change is a challenge’), that are in favour of policies that would reduce emissions (e.g., ‘We must fight climate change.’), or they may form part of specific proposals for policies that would reduce net^^[[2]](#footnote-3)^^ greenhouse gas (GHG) emissions. Typical examples of climate policy instruments include: emissions trading, carbon taxes, feed-in tariffs, low carbon energy quota schemes, bans on fossil fuel plants without CCS and emissions or fuel economy standards for cars (Bailey and Compston, 2013)^^[[3]](#footnote-4)^^. They may also include elements of a domestic framework for climate policy (e.g., climate legislation, new or more ambitious emissions targets, climate-specific institutions) (EBRD and GRI, 2011). Quasi-sentences that are ‘pro-climate policy’, may also include proposals to reverse anti-climate policy measures (see below). However, they may also include indications of support for a wide range of other policies that, if implemented, would reduce greenhouse gas emissions. The party may or may not explicitly link the policy proposal with emissions reductions. Note that climate policies may also include the *enhancement of carbon sinks* (e.g., wetlands).

**2. Anti-climate policy quasi-sentences**

These are pieces of text that *indicate support for policies that, if implemented, would increase net greenhouse gas emissions or reduce carbon sinks*.^^[[4]](#footnote-5)^^ These quasi-sentences may be statements that deny that climate change is a problem, general statements against policies that would reduce emissions (e.g., ‘We should not give in to climate change alarmism’) or (more commonly) they will be policy proposals that would increase net GHG emissions (Compston and Bailey, 2013: 147)^^[[5]](#footnote-6)^^. These proposals may include the reversal of climate policies (see above). They may also include a wide range of policies that have increased emissions as a side-effect. Compston and Bailey identify the following as examples of anti-climate policy: construction of, or approval/incentives for, new fossil fuel power stations; new or increased fossil fuel subsidies; new or increased subsidies for energy-intensive industries: iron and steel, non-ferrous metals, chemicals and fertilizers, petroleum refining, cement and lime, glass and ceramics, pulp and paper, food processing; new trade liberalization agreements; new or increased subsidies for the automotive, aerospace or shipping industries; construction of, or approval/incentives for, new airports; increased support for meat production; and action by state agencies to clear forests for farmland, or approval/incentives for this. For a wider range of examples, see Table 2 in Compston and Bailey (2013: 148). This table may be a useful (but not definitive) guide to identifying anti-climate policies.

Anti-climate policies can be more difficult to identify than pro-climate policies. Therefore, in addition to the general guidance provided by Compston and Bailey’s (2013) lists, we are developing a list of commonly encountered questions and answers so that we can maintain consistency between coders.

1. *Do all policies that would stimulate economic growth count as anti-climate policies?* Economic growth is linked to emissions (e.g., Stern, 2006: xi, xii). In order to maintain consistency across codings, we identify a) positive mentions of economic growth and b) proposals for general growth stimulus packages as anti-climate policies. However, we do not identify economic activity, job-creation, house-building, pro-business policies etc. (as such) as anti-climate policies. Mentions of “green growth” are pro-climate.
2. *Do low-tax policies count as anti-climate policies?* They count only if they are statements that could include opposition to a carbon tax. E.g., “We promise no new taxes” in a context where there is no carbon tax. However, specific promises on income tax or other types of tax do not count as anti-climate policies.
3. Likewise, *general statements against regulation* might cover regulatory climate policies and therefore should be coded as anti-climate policies.
4. *The promotion of international tourism* is coded as an anti-climate policy, although it is not identified by Compston and Bailey as such. We disregard tourism promotion that is explicitly domestic (non-international).
5. *Policies that might encourage population growth* (e.g., subsidised childcare) are not counted as anti-climate policies (contra. Compston and Bailey). We would argue that they are too widely diffused in manifestos and the effects of individual policies are too marginal to be coded.

Other guidelines:

- Some pieces of text (pro- and anti-climate policies) will require some further research to assess whether they would increase or reduce emissions. One example of this has been High Speed Rail in the UK (see Appendix to the coding instructions). You are welcome to submit similar notes with your coded data.
- Context (year and country) is important. Remember that we are examining whether text support policies that would increase (reduce) emissions in a specific context. For example, in an (hypothetical) energy system powered fully by coal, developing new nuclear capacity will clearly be a pro-climate policy; in an (hypothetical) energy system powered fully by renewables, developing new nuclear capacity would be an anti-climate policy.
- If in doubt, code the text as you think it should be coded, code it as ‘hcode’ (hard to code), enter a comment, and then discuss it with others in the project team.

**3. Neutral or ambiguous quasi-sentences**

Neutral quasi-sentences are not the same as quasi-sentences that are not sufficiently relevant to GHG emissions. A neutral (or ambiguous) quasi-sentence should be relevant to net GHG emissions, but its content should imply that net emissions would be maintained at current levels (e.g., by clearly displacing emissions nationally or internationally [see footnote on net emissions, above] or by including policies that both increase and reduce emissions).

**4. Quasi-sentences that are not sufficiently relevant**

These are quasi-sentences that are not sufficiently relevant to net greenhouse gas emissions to be coded as 1, 2 or 3 (e.g., ‘We are committed to improving language education in schools’). We identify these quasi-sentences (so that we can count them) but we do not need to enter ‘4’ in each instance (we can simply leave the cells blank for now).

**Summary of variables**

The key variables are as follows:

- Year
- Party.
- Qsno: Sequential numbers assigned to quasi-sentences.
- Qstext: “text” of the QS
- Hsplit: ‘hard split’ (0/1) – if you are unsure about whether the sentence should be split into quasi-sentences or not (see Froio 2012: 5).
- Junk (0/1) (see Froio 2012: 5)
- Head (0/1) (see Froio 2012: 5)
- Ccode: 1, 2, 3 or 4 (see above)
- Hcode (0/1): ‘hard code’ – if you have remaining doubts about some aspect of the coding that you have done (see also Froio 2012: 7).
- Comment: to help anyone who uses the data with resolving outstanding problems or understanding the coding.

**Appendix to Coding Instructions: High speed rail in the UK 2010 manifestos**

How should high speed rail content be coded for UK manifestos in 2001, 2005 and 2010?

1. The Government's March 2010 plan entitled *High Speed Rail* cited HS2’s estimate that the emissions impact would be between -25 and +26.6 million tonnes (Department for Transport, 2010: 53).

2. In 2010, the Climate Change Committee also suggested that it would be carbon neutral (Climate Change Committee, 2010: 185).

3. An NGO report in 2012 suggested that there would be savings in the 60 year timeframe (Greengauge, 2012) and in November 2013 the Department of Transport believed that it would produce carbon savings (Department of Transport, 2013).

4. A HS2 briefing (‘CS034’) published in November 2013 suggested that in the first sixty years of the project, savings would be less than emissions.

For the purposes of coding the manifestos, #1 and #2 are most important, as they reflected the available analysis at the time of the 2010 manifestos and they are the most authoritative sources. Note also that Greengauge is a pro-HS2 NGO. Therefore, I suggest coding high speed rail in the UK as ambiguous/neutral.

**References**

Bailey I and Compston HW (2013) Comparing Climate Policies: the Strong Climate Policy Index. Paper presented at the *Annual Conference of the Political Studies Association*.

Bernauer T and Böhmelt T (2013) National climate policies in international comparison: The Climate Change Cooperation Index. *Environmental Science & Policy* 25:196–206.

Burck J, Hermwille L and Bals C (2013) The Climate Change Performance Index: Background and Methodology. Report, Germanwatch, Bonn.

Climate Change Committee (2010) *The Fourth Carbon Budget*. Report, Committee on Climate Change, London.

Compston H and Bailey I (2013) Climate Policies and Anti-Climate Policies. *Open Journal of Political Science* 3(4):146–157.

Compston H and Bailey I (2016) Climate policy strength compared: China, the US, the EU, India, Russia, and Japan. *Climate Policy* 16(2):145–164.

Däubler T, Benoit K, Mikhaylov S and Laver M (2012) Natural Sentences as Valid Units for Coded Political Texts. *British Journal of Political Science* 42(4):937–951.

Department for Transport (2010) *High speed rail*. Report, Stationery Office, London.

Department of Transport (2013) *London- West Midlands environmental statement Volume 3 | Route-wide effects*. Report, HS2 Ltd., London.

EBRD and GRI (2011) *The Low Carbon Transition. Special Report on Climate Change.* Report, European Bank for Reconstruction and Development and Grantham Research Institute, London.

Froio C (2012) UK Policy Agendas Project -- French Agendas Project. Coding Party Manifestos. Version 2.0. Manuscript.

Greengauge (2012) *High-Speed Rail. The carbon impacts of High Speed 2*. Report, Greengauge 21.

Stern N (2006) *The Stern review on the economics of climate change*. Cambridge: Cambridge University Press.

**Online Appendix C. Coding subcategories.**

These are the categories that we use in the coding scheme to describe individual pieces of text. They were developed inductively. Initially, coders gave the text a primary label and a secondary label if appropriate. Where more than one label applied, final decisions on the primary label were based on which category was primary in a quasi-sentence or (if that did not differentiate between the elements) which was mentioned first. The categories listed below were constructed by merging multiple logically coherent subcategories (i.e., labels). Coding is context-specific (i.e., the same policy proposal may have a different effect on GHG emissions in different countries or at different times). The descriptions below indicate content that is *typically* included (and not included) in the subcategories and, thus in the overall pro-climate and anti-climate categories.

An asterisk* indicates a subcategory that is excluded for the calculation of Core measures.

*Pro-climate categories*

**Pro-environment.** *Pro-environment text that potentially includes climate policy.* Includes general statements in favour of environmental protection that may include the climate, pro-environmentalism, pro-sustainable development, pro-green growth, general criticisms of the government’s environment policy that potentially include but are not specific to climate policy, pro-general environmental EU action that potentially includes climate change, pro-use of environmental indicators, pro-foreign environmental aid, pro-environmental taxation, sustainable tourism.

**Pro-lower carbon energy.** *Pro-renewables and cleaner energy. Includes nuclear and includes gas distribution where this would be an improvement on the status quo in terms of GHG emissions.* The coding scheme is typically indifferent on privatization of the energy sector unless there is evidence that it would contribute to reduced GHG emissions. Inclusion of content in favour of international interconnections depends on context. It includes proposals on clean coal in the few instances where they occur, giving parties the benefit of the doubt regarding its feasibility and impact.

**Pro-energy efficiency.** *Energy efficiency measures.* Includes buildings’ energy efficiency. Includes smart grids and efficiency in transmission and generation. Includes measures to prevent water wastage during distribution or consumption.

**Pro-lower carbon transport**. *Pro-public transport, cycling and pedestrians, cleaner vehicles, road-pricing.* Includes general mentions of ‘sustainable transport’, pro-road pricing. Includes high-speed rail where there is evidence that it would reduce emissions. Safety (eg., rail safety) is not included. Establishment of transport regulators or chiefs not generally included unless specifically aimed at improved lower carbon transport. Anti-road congestion measures are not included. Rural transport schemes are included if they mainly imply public transport supports. Includes bus links to airports. Includes teleworking.

**Pro-carbon sinks.** *Pro-forestry, wetlands, protection of green areas.* Includes promotion of brownfield development instead of greenfield development.

**Pro-climate policy (other)**. *Content that has not been included in named categories in this table.* Includes acknowledgement of climate change as a problem; acknowledgement of climate change impacts; general statements in favour of emissions reductions and climate action; specific climate policy proposals (e.g., climate legislation, carbon tax, GHG emissions limits).

**Waste**.* *All pro-climate content at all stages of the waste cycle.* Includes all related content that indicates support for waste policies that would reduce GHG emissions or increase sinks.

**Planning**.* *Spatial planning, pro-urban living measures.*

**Agriculture and food.*** *Environmental protection measures in agriculture, aquaculture and forestry that include (or that may include) emissions-relevant measures*. Opposition to subsidies for GHG-intensive agriculture (including pro-CAP reform statements). Pro-domestic consumption of local and national food. Includes policies for better provenance labelling but do not include international trade promotion of local produce. Does not include organic farming.

**Anti-growth.*** Explicit anti-economic growth statements.

*Anti-climate categories*

**Anti-environmental taxes.** Includes text that is anti-environmental taxes, anti-carbon tax, anti-fuel tax, pro-lower carbon tax, opposition to increased environmental taxation or pro-additional exemptions to an environmental tax.

**Pro-aviation and shipping.** Includes support for the aerospace industry. Pro-shipping content is included where this does not clearly displace higher-carbon transport.

**Pro-roads.** Support for road-building. Support for buying cars and for the car industry. Road safety is not included.

**Anti-nuclear.** Does not include opposition to specific waste storage facilities or to specific facilities abroad (e.g., Sellafield in the Irish case).

**Pro-fossil fuels.** Pro-fossil fuel extraction and in favour of consumption where these are not ‘cleaner’ (i.e., less GHG-intensive) sources.

**Other anti-climate.** Content that has not been included in named categories above.

**Agriculture**.* *Text explicitly in favour of GHG-intensive agriculture.* Includes content in favour of the CAP and other subsidies for GHG-intensive farming. We code this conservatively; only explicit supports for GHG-intensive farming are coded.

**Pro-growth**.* *Explicit positive mentions of economic growth.* Only explicit positive mentions of ‘economic growth’. Also includes general growth-stimulus measures (e.g., general stimulus packages) and explicit pro-consumption mentions. Does not include economic growth in the context of least-developed countries.

**Anti-taxes.*** *Fewer or lower taxes.* General: must not be specific to one type of (non-climate relevant) taxation.

**Anti-regulation.*** *Less regulation.* General: must not be specific to one type of (non-climate relevant) regulation. And can be specific to environmental or climate regulation. Does not include general statements concerning national competitiveness.

**Pro-tourism.*** Does not include measures directed explicitly at domestic tourism. Does not include ‘sustainable tourism’, which is coded as pro-climate.

**Pro-global free trade.*** Support for global free trade regimes. Does not include specific pro-export or pro-trade content. International regime level: content that is simply in favour of national exports is not included. Global scope: EU single-market not included.

**Online Appendix D. Questionnaire.**

**Coding document 3: Document description questionnaire**

Which party, year and document are you answering for?

**Problem acknowledgement**

1. Does the document reject, ignore or acknowledge the problem of climate change? If it rejects or acknowledges climate change, then provide a typical example from the text in native language with an English translation.
2. Does the document acknowledge present or future impacts of climate change (e.g., on health, immigration, biodiversity, flooding etc.)? Which impacts does it acknowledge? This can include local, national and international impacts.

**Climate goals**

1. Does the document commit the party to general national climate goals (e.g., specific emissions levels at specific dates, carbon neutrality). Describe these goals. You can disregard specific goals on renewable energies or on sectoral emissions.

**Prominence/attention**

1. What was the title of the section that dealt (more than any other section) with climate change issues? Where was the section that dealt with climate change placed in the manifesto? Answer: section X of Y. And: begins on page X of Y (for this, use the overall number of pages in the pdf/paper document rather than the assigned page numbering).
2. Is climate change mentioned in the ‘front matter’ of the document? Which parts (cover, table of contents, foreword, introduction, leader’s introduction, other [describe]).
3. How often is “climate” (in a climate change-relevant context, excluding uses such as ‘political climate’ or ‘business climate’) or “global warming” mentioned in the document? What is the overall word count? Use the text file from polidoc.net to get an overall word count.

**Online Appendix E. Notes on data used for Tables 3 and 6.**

**Note on CMP data used in analyses described by Table 3.** *Forza Italia’s* values in the CMP for 2006 were compared with the *Casa delle Libertà* document in our data. For France in 1993, we used the mean value of the UDF and RPR in the CMP data. We compared the Italian *Ulivo*’s value in 1996 to the *Partito Democratico della Sinistra* in the CMP data.

**Note on expert survey data used in analyses described by Table 3.** Benoit and Laver’s (2006) measure refers to the importance of the issue for the party; CHES data refers to the ‘importance/salience’ of the issue for the party. Expert survey (and EU Profiler/EU&I data in the analyses that follow in Table 6 were paired with the closest election within 2.5 years either side of the time point. The time point for Benoit and Laver’s data is assumed to be mid-2003; CHES is assumed to be mid-2010 (and mid-2014 for the analyses in Table 6. For the *Ulivo* in 2001, we use the mean of DS and the *Margherita* in the Benoit and Laver survey; likewise, we use the mean of UDF and RPR for the French centre-right.

**Note on EU Profiler/EU&I data used in analyses described by Table 6.** The items used for the index are as follows: ‘The promotion of public transport should be fostered through green taxes (e.g. road taxing)’ and ‘Renewable sources of energy (e.g. solar or wind energy) should be supported even if this means higher energy costs’. The responses range from ‘Strongly agree’ to ‘Strongly disagree’ on a five-point Likert scale.

**Note on expert survey data used in analyses described by Table 6.** The CHES item is as follows: ‘0 = Strongly supports environmental protection even at the cost of economic growth; 10 = Strongly supports economic growth even at the cost of environmental protection’. The Benoit/Laver item is identical, with the exception of the word ‘strongly’, which it excludes. The correlations with the CHES data only (n=20) are very similar to the results in Table 6, albeit with higher *p* values.

**Online Appendix F. Report on results excluding Denmark.**

Document characteristics (cf. Tables 2 and 5): across the four sets of tests that we did (one for each measure), the mean differences between groups decline. They lose their significance only for one of our four measures (the General measure). In all instances, the differences continue to be in the expected direction.

Comparisons with existing measures of environment and climate policy preferences (cf. Tables 3 and 6): the results range from very similar results to those with the full data set (for the CMP, *n*=50); to higher correlation coefficients that lead to substantively similar conclusions (for the expert survey data, *n*=26); to similar correlation coefficients that lose significance (CAP Environment category [*n*=24], expert survey salience measures [*n*=24]; EU Profiler/EU&I positional measures [*n*=17]). The correlation coefficients between our positional measures and Lowe et al’s positional measure becomes considerably weaker (r = 0.2; n=40) and clearly non-significant. One set of results is substantively different: correlations with the CAP climate measure (*n*=24) loses significance and becomes negative (albeit weakly so). Both this and increased p-values associated with other variables may be accounted for by the low number of observations for which data is available in both the CAP and our data set.

In addition, the comparison of parties of the centre-left and centre right produces almost identical results and the mean difference between pre- and post-economic crisis documents remains similar, albeit losing its statistical significance.

1. Further discussions with coders established that this should be a reduction relative to a counterfactual situation in which the policy was not implemented (rather than a reduction *per se*). [↑](#footnote-ref-2)
2. ‘We specify net emission to exclude policies that simply shift emissions from one location [i.e., country] to another’ (Compston and Bailey, 2013: 147). In practice, this proved difficult to establish empirically, so we do not refer to net emissions in the main body of the paper. [↑](#footnote-ref-3)
3. Later published as Compston and Bailey (2016). [↑](#footnote-ref-4)
4. See fn.1. [↑](#footnote-ref-5)
5. Note that, in contrast to Compston and Bailey, we do not insist that this should be a proposal for policy *change*. An affirmation that a party will stick with a status quo policy that will increase emissions is, for our purposes, just as significant as a policy change that would have this effect. [↑](#footnote-ref-6)
