# Supplementary material for: Political parties and climate policy: A new approach to measuring parties’ climate policy preferences
Source: Party Politics. 2017 Mar 23;24(6):731–42. doi: 10.1177/1354068817697630 (PMC6201165; doi:10.1177/1354068817697630)
Supplement: Supplemental Material, CarterLadrechLittleTsagkroni_PP_Codebook - Political parties and climate policy: A new approach to measuring parties’ climate policy preferences [file CarterLadrechLittleTsagkroni_PP_Codebook.docx]

**Description of the Climate Change and Political Parties Dataset**

| Units | 64 parties at national elections |
| --- | --- |
| Number of Countries | 6 |
| Number of Elections | 32 |
| Time Period Covered | 1993-2015 |
|  |  |
|  |  |
| **Basic information** |  |
| Country | Country name |
| RL | Right- or left-of-centre |
| Party.name | Party name |
| Year | Election year |
| Crisis | Before or after mid-2008 |
|  |  |
| **Measures of climate policy preferences and their components** |  |
| Wordcount | Number of words in the document |
| Quasi.sentences | Number of quasi-sentences (QS) in the document |
| Pro.climate.QS | Number of pro-climate QS |
| Pro.climate.pct | Pro-climate QS as % of all QS |
| Anti.climate.QS | Number of anti-climate QS |
| Anti.climate.pct | Anti-climate QS as % of all QS |
| Core.pro.N | Number of Core pro-climate QS |
| Core.pro.pct | As % of all QS |
| Core.anti.N | Number of Core anti-climate QS |
| Core.anti.pct | As % of all QS |
| Position | Pro.climate.pct – Anti.climate.pct |
| Core.position | Core.pro.pct – Core.anti.pct |
|  |  |
| **‘Document attributes’ related to climate change** |  |
| See Appendix D for full questionnaire  Acknowledges | Acknowledges climate change as a problem |
| Goals | Commits to general national climate goals |
| Prominence.pages | After what proportion of the document does the section dealing with climate change appear? |
| Front.matter | Is climate change mentioned in the ‘front matter’ of the document? |
| Climate.mentions | Number of mentions of climate change and cognate terms |
| Mention.pct | (Number of mentions of climate change / wordcount)*100 |
|  |  |

| **Subcategories: pro-climate** |  |
| --- | --- |
| See Appendix C for further details of these subcategories. | |
| Pro.environment.QS | Number of pro-environment QS |
| Pro.environment | As a % of all pro-climate QS in the document |
| Pro.lower.carbon.transport.QS | Number of pro-lower carbon transport QS |
| Lower.carbon.transport | As a % of all pro-climate QS in the document |
| Pro.carbon.sinks.QS | Number of pro-carbon sinks QS |
| Pro.carbon.sinks | As a % of all pro-climate QS in the document |
| Pro.lower.carbon.energy.QS | Number of pro-lower carbon energy QS |
| Pro.lower.carbon.energy | As a % of all pro-climate QS in the document |
| Pro.energy.efficiency.QS | Number of pro-energy efficiency QS |
| Pro.energy.efficiency | As a % of all pro-climate QS in the document |
| Pro.climate.policy.other.QS | Number of pro-climate policy (other) QS |
| Pro.climate.policy.other | As a % of all pro-climate QS in the document |
| Agriculture.and.food.QS | Number of (pro-climate) agriculture and food QS |
| Agriculture.and.food | As a % of all pro-climate QS in the document |
| Anti.growth.QS | Number of anti-growth QS |
| Anti.growth | As a % of all pro-climate QS in the document |
| Waste.QS | Number of waste QS |
| Waste | As a % of all pro-climate QS in the document |
| Planning.QS | Number of planning QS |
| Planning | As a % of all pro-climate QS in the document |
|  |  |
| **Subcategories: anti-climate** |  |
| See Appendix C for further details of these subcategories. | |
| Anti.environmental.taxes.QS | Number of anti-environmental taxes QS |
| Anti.environmental.taxes | As a % of all anti-climate QS in the document |
| Pro.aviation.and.shipping.QS | Number of pro-aviation and shipping QS |
| Pro.aviation.and.shipping | As a % of all anti-climate QS in the document |
| Pro.roads.QS | Number of pro-roads QS |
| Pro.roads | As a % of all anti-climate QS in the document |
| Anti.nuclear.QS | Number of anti-nuclear QS |
| Anti.nuclear | As a % of all anti-climate QS in the document |
| Pro.fossil.fuels.QS | Number of pro-fossil fuels QS |
| Pro.fossil.fuels | As a % of all anti-climate QS in the document |
| Other.anti.climate.QS | Number of other anti-climate QS |
| Other.anti.climate | As a % of all anti-climate QS in the document |
| Agriculture.QS | Number of agriculture QS |
| Agriculture | As a % of all anti-climate QS in the document |
| Pro.growth.QS | Number of pro-growth QS |
| Pro.growth | As a % of all anti-climate QS in the document |
| Anti.taxes.QS | Number of anti-taxes QS |
| Anti.taxes | As a % of all anti-climate QS in the document |
| Anti.regulation.QS | Number of anti-regulations QS |
| Anti.regulation | As a % of all anti-climate QS in the document |
| Pro.tourism.QS | Number of pro-tourism QS |
| Pro.tourism | As a % of all anti-climate QS in the document |
| Pro.global.free.trade.QS | Number of pro-global free trade QS |
| Pro.global.free.trade | As a % of all anti-climate QS in the document |
|  |  |
|  |  |
| **Secondary data** |  |
| *Manifestos Research Group* | |
| Cmpenv501 | per501 Environmental Protection |
| Cmpposn | Environmental policy position calculated using Weale et al’s (2000) formula: per501 – per410 |
| X501qsCMP | Number of QS coded, per 501 |
|  |  |
| *Lowe et al. (2011) logged variables* | |
| Env_imp | log (per 501) |
| Altenv_logit | Inverted positional indicator (see Lowe et al. 2011) |
|  |  |
| *Comparative Agendas Project* | |
| Capenv | Category 7: Environment as % of document |
| Cap705 | Subcategory 705: ‘Air pollution, global warming and noise pollution’ as % of document |
| X7CAPqs | Number of QS coded, Category 7 |
| X705CAPqs | Number of QS coded, Subcategory 705 |
|  |  |
| *EU Profiler and EU and I* | |
| Climatetwo4 | An index of two climate policy items that are common to the EU&I and EU Profiler surveys: ‘The promotion of public transport should be fostered through green taxes (e.g. road taxing)’ and ‘Renewable sources of energy (e.g. solar or wind energy) should be supported even if this means higher energy costs’. The responses for each item range from ‘Strongly agree’ to ‘Strongly disagree’ on a five point Likert scale. |
|  |  |
| *Expert survey data* | |
| Expertsposition | Expert survey environmental policy position (CHESS and Laver and Benoit, common scale) |
| Salienceboth | Expert survey environmental policy salience (CHESS and Laver and Benoit) |
